# Knowledge and Utilisation of Iodised Salt by School Children and Food Vendors in Dar es Salaam, Tanzania

**DOI:** 10.24248/eahrj.v7i2.732

**Published:** 2023-11-30

**Authors:** Mario Sibamenya Venance, Judith Kimywe, Haikael D. Martin

**Affiliations:** a Department of Food Biotechnology and Nutrition Sciences, Nelson Mandela African Institution of Science and Technology, Arusha, Tanzania; b Division of Health, Social welfare, and Nutrition, Buhigwe District Council, Kigoma, Tanzania; c Department of Foods, Nutrition, and Dietetics, Kenyatta University, Nairobi, Kenya

## Abstract

**Background:**

Universal Salt Iodisation (USI) is globally accepted as a cost-effective strategy for controlling Iodine Deficiency Disorders (IDD). However, there is a paucity of data on the proper use of iodised salt among food vendors. Thus, the present study assessed the School food environments and iodised salt practices among school food vendors in Tanzania.

**Methods:**

A cross-sectional study was conducted involving 322 school children and 30 school food vendors. A stratified multistage followed by a systematic random sampling technique was used to recruit schools and children. Salt samples were collected from food vendors and analyzed using the iodine rapid field test kit and then analyzed quantitatively using an iodometric titration method. The data on knowledge, attitude, and practices was collected using customized Iodine deficiency-related questionnaires recommended by FAO to assess iodine deficiency-related factors. For school food environments, 3 tools were designed and used to get information; the teachers, students, and school food vendors' tools. A chi-square test was used to establish an association between variables.

**Results:**

About 76.7% of the salt samples from school food vendors were adequately iodised (≥15 ppm) with the mean iodine content of 39.26 ± 11.06. More than half (70%) of the food sold around school compound were junk food. Half (63.3%) of the food vendors had poor practices of iodised salt utilisation.

**Conclusion:**

A substantial achievement has been made in awareness creation of the importance of using iodised salt. Nonetheless, there is a limited knowledge on salt handling practices including discretionary salt use exacerbated by poor school food environment. To optimally eliminate IDDs, the government should now place more emphasis on proper salt handling practices from the production site, food handlers, and much more to consumers. School children should be encouraged on healthy eating habits, by improving school feeding programs and change the current food environments in schools.

## BACKGROUND

Iodine is a vital micronutrient that is hard to get through normal foods in the body. This is because the concentration of iodine in foods depends on the amounts contained in the soil.^1,2^ A wide range of life-threatening conditions termed *Iodine Deficiency Disorders* (IDD) arise in inadequate intake. In fetal life and early childhood hood, severe Iodine Deficiency (ID) could contribute to irreversible brain damage, abortion, stillbirth, low child IQ, and cretinism.^3,4^ More than 285 million children and 2.7 billion people worldwide are at risk of iodine deficiency.^5^ World Health Organization (WHO) acknowledges ID as a global issue.^6^ In Tanzania, 41% of the population is at risk, with ID attributed to more than 30% of infant and child mortality.^7^

WHO recommends Universal Salt Iodisation (USI) for control interventions of IDDs.^6^ Globally, household coverage using iodized salt exceeds 70%. However, country-by-country household salt distribution varies widely in Africa. Based on data from 2007 to 2011, only 8 countries achieved the USI target, and 11 had iodine deficiency. Sudan, Ethiopia, Angola, Algeria, Morocco, Mozambique, and Ghana have the most significant burden of iodine deficiency in Africa.^8^

Tanzania is improving but far from a promising country for USI target achievement.^8^

In Tanzania, iodized salt utilisation showed a marked increase from 47% in 2010 to 61% in 2016. Anomalies in the utilization of iodized salt have been identified with socioeconomic status varying from 96% in the most affluent homes to 69% in the most economically disadvantaged families. Iodized salt utilization is above 90% in Arusha, Mbeya, and Dar es Salaam and lowest in Mtwara (27%), Lindi (23%), Simiyu (20%), and Kaskazini Pemba (14%). As a result, goitre affected 12.3% of school-age children.^9^

Since 1990, the Tanzanian government has strengthened and initiated USI initiatives and envisaged a strategy for achieving a practical elimination of IDDs through universal salt iodisation. Still, only 61% of households use adequate iodised salt.^9^ Indeed, IDD remains the most significant public health issue across all populations.^6^ Evidence from several studies found that enhancing public knowledge and public education on iodine among individuals is one of the most significant contributors to the successful control of iodine deficiencies.^10,11^

Iodine deficiencies mostly arises from 2 main factors: insufficient iodine intake through the diet and impaired absorption inside the human body.^12^ The primary dietary sources of iodine with the highest concentration are iodised salt, seaweed and shellfish.^13,14^ Additionally, foods derived from animals, such as meat and milk, can also serve as a substantial supply of iodine, particularly if the animals have been grazing on soils that are rich in iodine.^15^ Since the major source of dietary iodine is through iodised salt, proper use of iodised salt through discretionary salt use is very important especially among school children

Schools are intended to protect kids' brains and bodies. As learning institutions, schools may promote healthy or unhealthy eating within and outside the school.^16^ School is where kids spend most of their time and eat. School lunch programs are vital for low-income pupils. Supplying and encouraging unhealthy foods in and near schools promotes childhood obesity and malnutrition particularly micronutrient deficiency.^16^ Strong school food policies that restrict access to unhealthy foods; protect kids from food industry marketing, and emphasize the nutritional standards for school children who need to grow, develop, and succeed; will encourage kids to eat healthier at school and homes.^17^

Apparently, information on school food environments and iodised salt practices among school food vendors in Tanzania are limited while no studies have been reported among food vendors. Thus, this study offers fundamental data that can serve as a foundation for future quantitative research focused on examining the extent to which school food environment and food vendors in Tanzania contribute to iodine and other micronutrient intakes. Therefore, this study aimed to assess the school food environments and iodised salt practices among school food vendors in Dar es Salaam, Tanzania.

## METHODS

### Study Design and Settings

A school based cross-sectional study was conducted in Kinondoni, Tanzania. Kinondoni is one of the 5 municipalities within Dar es Salaam region. The municipal is administratively divided into 2 divisions, 20 wards, and 106 hamlets. It has a total population of 1,775,049 (914,247 females and 860,802 males), with 446,504 households and four people per household on average.^18^ The municipal has 121 primary schools and 72 secondary schools. It was purposively selected because of its high proportion of urban population together with reasonably high GDP per capita.^19^ These factors are known to be connected to increased access to food intake away from home. The study was conducted from May 8 to August 3, 2019. The study participants were school children and food vendors who live in the Kinondoni municipality. School children of any sex aged between 6—14 years were included in the study. The study also included all food vendors who sold food around school compounds for at least six months at the time of the study.

### Sample Size and Sampling Procedure

The sample size for school children was determined, as suggested by Pourhoseingholi et al.^20^ The estimated sample generated was based on the previous prevalence of adequate iodised salt of 61%, marginal error of 5%, and a confidence interval of 95%, which gives n = 365. The sample size for food vendors was 36, and this included all listed food vendors around the school compound: making a total of 401 sample population. A stratified multistage sampling technique was used in selecting participants for recruitment. 5 municipal wards were initially stratified into high and low socioeconomic status (HSS and LSS). Then, 8 government schools from the 5 wards were randomly selected. A-List of all pupils aged 6–14 years enrolled in the schools was identified from the schools' attendance registry. Then, a systematic random sampling technique was used to select the required sample.

### Data Collection and Quality Control Procedures

5 trained Nutritionists gathered information regarding knowledge and practice of iodised salt utilisation and their associated factors using pretested questionnaires. The questions were designed based on the more extensive study's research objectives. More information on children's knowledge, attitude, and dietary habits toward healthy eating were obtained through Focus Group Discussion (FGD). In each school, one FGD was conducted and consisted of 8 children aged 6—7 years who were not part of the written questionnaires. The principal investigator and supervisors ensured the accuracy and completeness of the daily collected data. Any discrepancies were addressed before continuing with the data collection the next day.

### Salt Collection and Analysis

Samples of salt were collected from school food vendors in order to conduct an analysis for iodine content. Every school food vendor was asked to provide 2 teaspoons of salt in a clean and airtight plastic pouch. The salt collected was that currently used by food vendors. The iodine content of each salt sample was qualitatively assessed using the MBI Rapid Test Kit.^6^ The kits were comprised of a solution based on stabilized starch. A single droplet of this solution was applied onto a teaspoon of salt, and the resulting iodine content was assessed using a color chart. The iodine levels were then classified and expressed in parts per million (ppm), with levels below 15 ppm considered sufficient, levels between 15 and 30 ppm categorized as medium, and levels at 0 ppm indicating the absence of iodine.^6^ Additionally, a quantitative analysis was conducted on the salt obtained from school food vendors employing the iodometric titration technique.^6^

### Assessment of Knowledge and Practice of Iodised Salt by Food Vendors

The respondents' awareness and practice of iodised salt use were measured using several questions. Among the questions asked were: Benefits of iodised salt, Iodine rich foods, IDDs, salt storage place, the time when salt is added during food preparation, favourite child foods, as well as their understanding of balancing diet. For each correct answer, 2 marks were awarded and one for each wrong answer. Scores below and above the mean were later categorized as “good and poor knowledge” and ``good and poor practices of iodised salt use," respectively

### Assessment of the School Food Environment and Anthropometric Measurement

The evaluation of the school food environment involved the utilisation of a modified standardized checklist, as proposed by Mckinnon et al.^21^, which encompassed a series of interrogative items. The checklists encompassed a compilation of food items that conform to specific predetermined criteria for evaluation, such as categorizing them as either unhealthy food or healthy food. The inclusion of the food items in the aforementioned list serves as a clear indication of the prevailing school food environment.

The anthropometric measurements were carried out in accordance with established protocols as outlined by Cogill (2003). The measurement of body weight was conducted using an SECA^™^ digital scale with a precision level of 0.1 kg. The measurement of height was conducted using a SHORR^™^ 2-piece height board with a precision of 0.1 cm. The participants' weight was measured while wearing a lightw8 garment and without footwear. The WHO AnthroPlus software was employed to assess the measurements of height and weight, and afterwards calculate the z scores for body mass index (BMIZ) based on age and sex.

### Ethics Approval and Consent to Participate

Ethical approval for the study was given by the Northern Tanzania Health Research Ethics Committee (Certificate number KNCHEC0012). Permits were obtained from the Municipal primary education officer. The study was introduced to students one day before data collection. Pupils were given consent forms with complete research details to present to their parents. The student submitted the completed consent documents the next day. On data collection day, parents' consent forms were reviewed for consent. Children whose parents or legal guardians refused research participation were omitted. For food vendors, written and oral consent was obtained after explanation of the study objectives and their need to participate. Participants were allowed to withdraw from the study at any time. Data privacy was also assured by the use of numbers for identification instead of names.

### Statistical Data Analysis

Data was tested, coded, and entered into Microsoft Excel and exported to SPSS 23 (IBM) for further analysis. Descriptive statistics are portrayed as numbers and percentages for categorical variables and continuous variables as mean and standard deviation. A chi-square test was used to establish an association between variables. Consideration for statistical significance was set at *P* < 0.05.

## RESULTS

### Socio-Demographic Characteristics

The study included 401 participants, of which 365 were school children and 36 food vendors. Of the children, 322 (88.2%) participated in the survey, while 30 (83.3%) of the food vendors responded to questionnaires. Of the food vendors, 25 (83.5%) were female, and the average mean age was 33.3 ± 8.8 years. Of school children, 51.9% were girls. The average mean age (years), weight (kg), height (cm), and BMI age were 17.3 ± 2.7, 34.5 ± 8.8, 140 ± 11.5, and 17.3 ± 2.7, respectively. The mean iodine content of the food vendor's salt was 39.26 ± 11.06 (Table 1).

**TABLE 1:** Population Characteristics by Socioeconomic Status in Kinondoni Municipality, Tanzania

Variables	Total	HSS	LSS	P value
Gender				
Boys (n; %)	158; 49.1	70; 44.3	88; 55.7	.304^a^
Girls (n; %)	164;51.9	67; 40.9	97;59.1	
Age (year; mean ± SD)	11.12 ± 2.37	10.94 ± 2.35	11.25 ± 2.38	.249^b^
Age group (n; %)				
6–8 years old	56; 17.4	24; 42.9	32; 57.1	.01^a^
9–11 years old	90; 28	52; 57.8	38; 42.2	
12–14 years old	176; 54.7	61; 34.7	115; 65.3	
W8 (kg; mean ± SD)	34.24 ± 8.84	34.25 ± 9.25	34.24 ± 8.48	.995^b^
Height (cm; mean ± SD)	140.0 ± 11.55	139.62 ± 11.402	140.28 ± 11.68	.610^b^
BMI (kg/m2; mean ± SD)	17.26 ± 2.72	17.32 ± 2.87	17.22 ± 2.62	0727
Family size				
≤ 5 family members	157;59	79;41.8	110;58.2	.417^a^
> 5 family members	109;41	58;43.6	75;56.4	
Family head				
Father	184;33.9	102;46.4	118;53.6	.108^a^
Mother	58;9.7	22;32.4	46;67.6	
Other	24;4.3	13;38.2	21;61.8	
Salt-Iodine levels from food vendors (ppm; mean ± SD)			39.26 ± 11.06	
Food vendor's characteristics (n=30)				
Gender				
Male (n; %)	5; 16.7	2; 11.1	16; 89.9.	304^a^
Female (n; %)	25; 83.3	5; 16.7	25; 83.3	
Age (year; mean ± SD)	33.3 ± 8.8	30.08 ± 6.37	35.5 ± 9.68	.1^b^

a Chi-square

b One-way ANOVA

### Food Vendor's Awareness of Nutrition, Health, Iodine-rich Foods, and Iodised Salt

Although 93.3% of food vendors are aware of iodised salt, the majority of them (60.0%) were unable to identify problems related to iodine deficiency and other iodine-rich foods (Table 2). The overall prevalence of participants' knowledge was 53.7%. The majority are aware of a balanced diet (53.3%). Radio and Television (50%) are their primary source of information. The type of food that these food vendors sold most to school children is junk food (70%) (Table 2).

**TABLE 2:** Knowledge of Food Vendors on Nutrition, Health, and Iodized Salt (N=30)

Variables	Category	Frequency	Percentage
Knowledge	Good	16	53.3
	Poor	14	46.7
Awareness on health nutrition	No	14	46.7
	Yes	16	53.3
Awareness on balanced diet	No	14	46.7
	Yes	16	53.3
A food selling most to the child	Junk foods	21	70
	Healthy foods	9	30
Awareness of iodine-rich foods	No	18	60
	Yes	12	40
Awareness on iodized salt	No	2	6.7
	Yes	28	93.3
Source of information on Iodized salt (n=28)	Radio	7	25
	TV and Radio	14	50
	TV	2	7.1
	School	5	17.9
Awareness on IDD	No	19	63.3
	Yes	11	36.7
Believed every salt contains iodine	Yes	9	30
	No	14	46.7
	Do not know	7	23.3
Believed every salt contains iodine	Yes	9	30
	No	14	46.7
	Do not know	7	23.3
Importance of iodized salt in the diet	Very important	11	36.7
	Somewhat important	15	50.0
	Not at all important	3	10.0
	No response	1	3.3

* Junk foods: Are energy-dense foods with high fat/sugar/salt content and low nutrients value in fiber, protein, mineral content, and vitamin content. Examples are potato chips, fried cassava, kachori popcorn, and the like.

** Healthy foods: Are nutrient-dense foods with low fat/sugar/salt content and high nutrients value in fiber, protein, mineral content, and vitamin content. Examples are fruits and vegetables

### School Children's Awareness of Nutrition, Health, Iodine-rich Foods, and Iodised Salt

School children aged 9—14 years (n= 266) were asked various questions on nutrition, health, iodine-rich foods, and iodised salt. Most of them, 201 (75.6%), mentioned Chips, Fried cassava, and Kachori (a spicy snack, made up of flour, salt, and oil) as their favorites. Only 38 children (14.3%) were able to mention iodine among the 3 essential trace elements they were asked for. Few 38 (25.6%) were able to say IDD was among the nutritional deficiency disorders they know.

Moreover, only 87 (32.7%) know that iodine content reduces when salt is in unenclosed containers. It was also noted that 149 (56%) of the children added salt after tasting the food (Table 3).

**TABLE 3:** Population Characteristics by Socioeconomic Status in Kinondoni Municipality, Tanzania

Variables	Category	Frequency	Percentage
Knowledge	Good	124	46.6
	Poor	142	53.4
Ever taught about nutrition	No	38	14.3
	Yes	228	85.7
Favourite food	Healthy foods	65	24.4
	Junk foods	201	75.6
Reasons for favorite	Availability	5	1.9
	Healthy	54	20.3
	Savoury	120	45.1
	Taste good/sweet	63	23.7
	No reasons	24	9.0
Mentioned iodine among 3 important minerals	No	228	85.7
	Yes	38	14.3
Mentioned IDD among nutritional deficiency disorders	No	198	74.4
	Yes	68	25.61
Salt is a good source of iodine	No	198	70.7
	Yes	68	15.8
	Do not know	198	13.5
All salt contains iodine	No	39	14.7
	Yes	183	68.8
	Do not know	44	16.5
Salt is a good source of iodine	No	198	74.4
	Yes	68	25.6
Iodine content reduces when salt is left uncovered	No	104	39.1
	Yes	87	32.7
	Do not know	75	28.2
Adding salt to food	Generally, add salt to food without testing	45	17.0
	Taste the food and generally add salt	149	56.0
	Taste the food but occasionally add salt	16	6.0
	Rarely or never add salt to the table	53	20.0
	Rarely/Never	3	1.0

### Utilisation and Availability of Adequately Iodised Salt among Food Vendors

The proportion of good practices in using iodised salt among food vendors was 36.7%. Of the salt samples, 76.7% were adequately iodised (Table 4). Only 5 (16.7%) of food vendors reported buying salt based on whether it contained iodine. The majority buy any salt brand without considering any factor. Only 12 (40%) of food vendors store salt in a container with a lid. Importantly, only a few of them 10 (33.4%) add salt at the end during cooking as recommended (Table 4).

**TABLE 4:** Availability and Practices of Using Iodized Salt among Food Vendors

Variables	Category	Frequency	Percentage
Salt iodine content (ppm)	≥ 15	23	76.7
	< 15	7	23.3
Practices (n=30)	Good	11	36.7
	Poor	19	63.3
Factors considered in buying salt	Iodized/Non-Iodized	5	16.6
	Price	2	6.7
	Brand	2	6.7
Salt brand used	Any salt	21	70
	Brand A	22	73.3
	Brand B	3	10
	Brand C	5	16.7
Type of salt used (n=30)	Iodised packed salt	30	100
	Coarse salt (Non packed)	0	0
Salt storage material	With closed container	12	40
	Without closed container	18	60
Addition of salt in cooking during (n=30)	Beginning	4	13.3
	Middle	16	53.3
	At the end of cooking	10	33.4
How often do pupils Ask for additional salt (n=30)	Always	2	6.7
	Often	20	66.7
	Sometimes	5	16.6
	Rarely/Never	3	10

### Factors Associated with Knowledge and Practice of Iodised Salt Utilisation among Food Vendors

The mean score for knowledge questions was 15.6 ± 4.2, and that of practice was 11.2 ± 0.9. Knowledge regarding iodised salt consumption was significantly associated with socioeconomic status, gender, age group, educational level, and marital status (*P* < 0.05). The proportion of proper use of iodised salt among food vendors was 36.7%. Proper use of iodised salt among food vendors who sell food to schools located in slum areas was significantly lower than their counterparts (*P* < 0.05) [Table 5]. There were significant associations between school socioeconomic status, Age groups, and salt iodine content (*p* < 0.005) [Table 5].

**TABLE 5:** Association of Knowledge and Use of Iodized Salt among Food Vendors (N=30)

Variables	Category	Knowledge				Practice			
		Good (n; %)	Poor (n; %)	χ^2^	P	Good (n;%)	Poor	χ^2^ (n; %)	P
Sodoeconomic status	HSS	11; 91.7	1; 8.3	11.8	.001	9; 75	3; 25	12.7	.000
	LSS	5; 27.8	13; 72.2			2, 11.1	16; 88.9		
Gender	Male	5; 100	0; 0	5.3	.022	1; 20	4; 80	0.7	.397
	Female	11; 44	14; 56			10; 40	15; 60		
Age group	<30 years	11; 91.7	1; 8.3	11.8	.001	6; 50	6; 50	1.5	.216
	>30 years	5; 27.8	13; 72.2			5; 27.8	13, 72.2		
Educational level	Non	0; 0	2; 100	8.2	.016	0; 0	2; 100	1.3	.524
	Primary	6; 37.5	10; 62.5			6; 37.5	10; 62.5		
	Secondaisr	10; 83.3	2; 16.7			5; 41.7	7; 58.3		
Marital status	Married	4; 33.3	8; 66.7	10.4	.015	2; 16.7	10; 83.3	3.8	.289
	Widowed	1; 50	1; 50			1; 50	1; 50		
	Divorced	1; 20	4; 80			2; 60	3; 40		
	Single	10; 90.9	1; 9.1			6, 45.5	5; 45.5		
Sells per day in TZS	1ess than 5,000	0; 0	2; 100	3.1	.218	0; 0	2; 100	1.7	.458
	Between 5k&10k	3; 75	1; 25			1; 25	3; 75		
	Above 10,000	13, 54.2	11; 45.8			10; 41.7	14; 58.3		

## DISCUSSION

The most effective and sustainable strategy for IDD control is the iodization of salt used in all food materials for both humans and animals.^6^ Accordingly, reinforcing salt iodization programs and improving the control and monitoring system is a critical step in eradicating the problem.^6^ Total eradication is achieved if more than 90 percent of households are using iodized salt.^6^ Nevertheless, food prepared or purchased outside the home is becoming an increasingly important component of the diet in many countries, especially in urban areas. The present study, therefore, included salt used by food vendors. It was then found that 76.6% of food vendor were using adequately iodized salt. The results are lower than the national and global target of achieving more than 90%, which signals the government to regularly monitor the quality of iodised salt from production sites to food retail outlets.

Findings revealed that the availability of adequately iodised salt in Kinondoni municipality (76.6%) is higher compared to other urban districts of Tanzania: In the sixteen regions with previously endemic goiter^23^, Arumeru (26.1%) and Ludewa (28.9%)^24,25^ reported lowest amounts. Similarly, these findings are significantly higher than a study in Northwest Ethiopia (26.2%).^26^ This is possibly due to increased efforts by the government to ensure the availability of iodised salt. The officials also put particular emphasis on promoting iodised salt's health benefits through local media to convey the required iodine awareness to the public.^27^ However, the availability of adequately iodised salt among food vendors (76.7%) is significantly lower than that of households in Iringa Tanzania (95%)^28^ and in Nigeria (97.5%).^29^ Vendors' poor practices towards iodised salt use and without using a salt container with a lid by 60% might expound the difference. These results further explain the need to include food vendors in future iodine national surveys for optimal iodine nutrition status of a population.

This study found that half (53.3%) of food vendors had good knowledge of iodised salt and other health and nutrition issues. The finding was lower than the 92.6% reported from the Southern Highlands of Tanzania.^28^ The variation might be due to the nature of study settings in that the previous study focuses on the areas previously identified as endemic iodine regions. These areas received a great deal of support from government and non-governmental organizations, where respondents had more opportunities to increase their exposure through media promotion of iodised salt than their counterparts. Different in study participants, households against food vendors in the present study could also explain the discrepancy. Information regarding iodine nutrition knowledge among food vendors is lacking in Tanzania and elsewhere despite its contribution to meal provision to many people. Excluding these groups in national surveys and local studies could result in missing out on essential and useful information for optimizing the iodine nutrition status of the population. The present findings explored their general understanding of iodised salt, health, and nutrition; and were lower compared to studies in households. Thus, interventions pertaining IDDs control programs should also focus on this group.

Integrating nutritional issues in primary school curricula would promote the acquisition of nutritional knowledge in different ways and the development of healthy eating habits in children.^30^ Imparting nutrition knowledge to this age group is vital for their future nutritional outcome.^31^ The present study examined the school food environments and the awareness of school children in nutrition, health, iodine-rich foods, and iodised salt. More than half (75.6%) of the food sold around the school compound were junk foods and only 46.6% had good knowledge of nutrition, health, iodine-rich food, and iodised salt based on the questions asked. This study is aligned with that done in Kenya, whereby pupils had moderate nutrition knowledge.^32^ Despite differences in the type of item between the 2 studies, the findings show that most school children are unaware of nutrition issues. For instance, in this study, 85.7% of the pupils acknowledged had been taught about nutrition in schools. Surprisingly only a few (14.3%) were able to mention iodine among the 3 essential trace elements. This shows that iodine is not among the most known essential trace elements to them. Little knowledge of micronutrients, particularly iodine among school children, can explain the persistence of micronutrient deficiency in Tanzania. More awareness creation on the importance of micronutrients is therefore needed in this young generation for their future nutrition outcome.

Besides, few 38 (25.6%) were able to mention IDD among the nutritional deficiency disorders they know. Moreover, the minority know if iodine content reduces when salt is in unsealed containers 87 (32.7%). Thus, a need for awareness creation as recommended by the WHO. Establishing school nutritional clubs could give the platform for the pupils to discuss various health and nutrition issues such as healthy eating and its associated benefits.

The accessibility, affordability, desirability and convenience of various foods are influenced by the food environment. A conducive school food environment promotes and motivates the school community, including children, school staffs and family to make dietary choices that align with healthier diets and enhanced overall well-being.^33^ In this study, 255 (75.5%) and other studies^31, 32, 34, 35^ reveal that the majority of the pupils eat junk foods than healthy foods while at school. Consumption of junk foods is now shown in an increasing rate of childhood obesity in Tanzania and hence needs intervention.

Governments possess many means through which they can influence the school food environments in order to foster healthier dietary choices and enhance nutritional standards. Several strategies can be implemented to promote healthier eating habits among children. These strategies encompass the establishment and enforcement of nutrition standards for food provided in schools, including meals and snacks. Additionally, making nutritious food more economically accessible through subsidies can also be effective.^36^ Furthermore, limiting the sale and advertisement of food products that are high in fat, sugar, or salt can contribute to the promotion of healthier dietary choices.

Proper use of adequately iodised salt is critical in IDDs control interventions. Nevertheless, the appropriate practice of using iodised salt in this study was significantly lower (36.7%) compared to the global and national target of 95%.^6^ As per recommendation, adequately iodised salt should be applied after cooking has been completed.^37^ Nevertheless, it is noted in this study that, the majority add salt in the middle (53.3%) while few at the end (33.3%) during cooking. The study is aligned with another study done in Northwest Ethiopia, whereby only 25.7% correctly use iodised salt that is at the end of the cooking period.^38^ It is now time to consider food vendors in future IDDs control interventions by creating awareness of the proper use of iodised salt. Another malpractice for proper utilisation observed is the on-table salt addition. The majority of the food vendors claimed that pupils usually ask for additional salt when eating particularly chips and fried cassava that, however, are the most preferred food to them. In areas where the salt used had high amounts of iodine than recommended, it could easily lead to excess iodine intake.^39^

In this study, in contrast to those schools with high socioeconomic status, respondents from schools with low socioeconomic status were more likely to have inadequate knowledge. This could entail improved literacy and access to the media among people with better socio-economic status, which could increase their understanding of the nutrition aspects and access to information, respectively. Further, people with poor socioeconomic status may tend to use non-iodised salt because they considered it cheaper and more effective than iodised salt.^40^

## CONCLUSION

The findings showed that the nutritional practices of school children and the types of food items sold in the school compound by food vendors were unhealthy for the growth and development of children. Pupils and food vendors had poor knowledge of nutrition and iodised salt practices and utilisation. The substantial achievement in awareness creation of the importance of using iodised salt is observed through the proper use of iodised salt, which remains low. The provision of nutrition education to the pupils and teachers at schools would enrich their knowledge and insights about healthy eating practices. The schools should have clear policies on food items served through the school feeding program and the type of food sold within school compounds. The presence of unhealthy foods on school premises should not be deemed acceptable.
